# 2-Amino-4-(2-chloro­phen­yl)-5,10-dioxo-5,10-dihydro-4*H*-benzo[*g*]chromene-3-carbonitrile

**DOI:** 10.1107/S1600536808039986

**Published:** 2008-12-03

**Authors:** Jinpeng Zhang, Xiaohong Zhang, Shu Yan, Ning Ma, Shujiang Tu

**Affiliations:** a Department of Public Health, Xuzhou Medical College, Xuzhou 221000, People’s Republic of China; b School of Chemistry and Chemical Engineering, Xuzhou Normal University, Xuzhou 221116, People’s Republic of China

## Abstract

In the mol­ecule of the title compound, C_20_H_11_ClN_2_O_3_, the pyran ring adopts a flattened-boat conformation. In the crystal structure, inter­molecular N—H⋯N and N—H⋯O hydrogen bonds generate edge-fused *R*_2_^2^(12) and *R*_2_^2^(14) ring motifs; the hydrogen-bonded motifs are linked to each other, forming a three-dimensional network. A π–π contact [centroid-to-centroid distance = 3.879 (3) Å] between the chloro­phenyl rings may further stabilize the structure.

## Related literature

For background to the biological activity of pyran and naphthoquinone compounds, see: El-Agrody *et al.* (2000 ▶); Mohr *et al.* (1975 ▶); Banzatti *et al.* (1984 ▶); Hatakeyama *et al.* (1988 ▶); Tandon *et al.* (1991 ▶); Kongkathip *et al.* (2003 ▶). For bond-length data, see: Allen *et al.* (1987 ▶). For puckering parameters, see: Cremer & Pople (1975 ▶). For hydrogen-bond motifs, see: Bernstein *et al.* (1995 ▶).
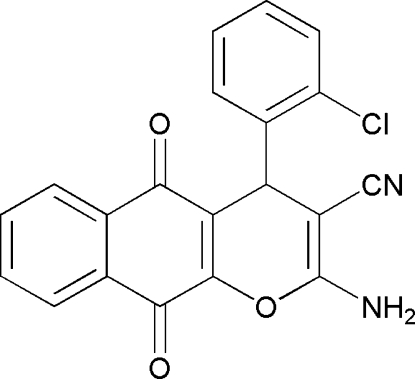


## Experimental

### 

#### Crystal data

C_20_H_11_ClN_2_O_3_
*M* *_r_* = 362.76
Triclinic, 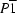

*a* = 8.3201 (10) Å
*b* = 9.3729 (12) Å
*c* = 11.0081 (16) Å
α = 93.015 (1)°
β = 96.393 (1)°
γ = 110.732 (2)°
*V* = 793.95 (18) Å^3^
*Z* = 2
Mo *K*α radiation
μ = 0.27 mm^−1^
*T* = 298 (2) K
0.17 × 0.15 × 0.10 mm

#### Data collection

Bruker SMART CCD area-detector diffractometer
Absorption correction: multi-scan (*SADABS*; Sheldrick, 1996 ▶) *T*_min_ = 0.956, *T*_max_ = 0.974
4207 measured reflections
2746 independent reflections
1566 reflections with *I* > 2σ(*I*)
*R*_int_ = 0.025

#### Refinement

*R*[*F* ^2^ > 2σ(*F* ^2^)] = 0.053
*wR*(*F* ^2^) = 0.100
*S* = 1.04
2746 reflections
235 parameters
H-atom parameters constrained
Δρ_max_ = 0.40 e Å^−3^
Δρ_min_ = −0.26 e Å^−3^

### 

Data collection: *SMART* (Bruker, 1997 ▶); cell refinement: *SAINT* (Bruker, 1997 ▶); data reduction: *SAINT*; program(s) used to solve structure: *SHELXS97* (Sheldrick, 2008 ▶); program(s) used to refine structure: *SHELXL97* (Sheldrick, 2008 ▶); molecular graphics: *SHELXTL* (Sheldrick, 2008 ▶) and *PLATON* (Spek, 2003 ▶); software used to prepare material for publication: *SHELXTL* and *PLATON*.

## Supplementary Material

Crystal structure: contains datablocks global, I. DOI: 10.1107/S1600536808039986/hk2585sup1.cif

Structure factors: contains datablocks I. DOI: 10.1107/S1600536808039986/hk2585Isup2.hkl

Additional supplementary materials:  crystallographic information; 3D view; checkCIF report

## Figures and Tables

**Table 1:** Hydrogen-bond geometry (Å, °)

*D*—H⋯*A*	*D*—H	H⋯*A*	*D*⋯*A*	*D*—H⋯*A*
N1—H1*A*⋯N2^i^	0.86	2.26	3.080 (4)	159
N1—H1*B*⋯O3^ii^	0.86	2.22	2.889 (3)	134

Symmetry codes: (i) 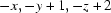; (ii) 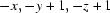.

**Fig. 1.**
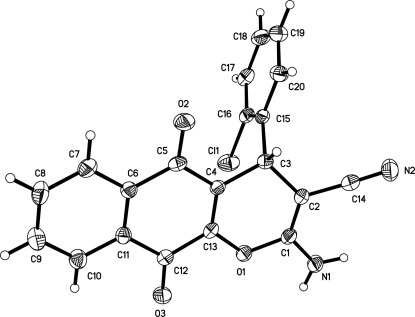
The molecular structure of the title molecule, with the atom-numbering scheme. Displacement ellipsoids are drawn at the 40% probability level.

**Fig. 2.**
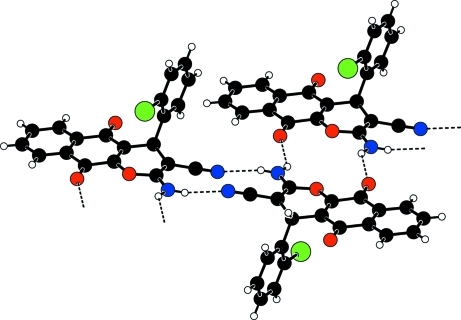
A partial packing diagram of the title compound. Hydrogen bonds are shown as dashed lines.

## supplementary crystallographic information

### Comment

Pyrans and their derivatives are important compounds, which are found to possess antibacterial (El-Agrody *et al.*, 2000) and antitumor (Mohr *et al.*,  1975) activities and antiallergic (Banzatti *et al.*,  1984; Hatakeyama *et al.*,  1988) and hypotensive (Tandon *et al.*,  1991) effects. Compounds of 1,4-naphthoquinone series possess potent and versatile biological activities, such as antiallergic and anticancer activities (Kongkathip *et al.*, 2003). For these reasons, 1,4-pyranonaphthoquinone derivatives possessing both pyran ring and 1,4-naphthoquinone motif are strongly desired. We report herein the crystal structure of the title compound.

In the molecule of the title compound (Fig. 1) the bond lengths (Allen *et al.*,  1987) and angles are within normal ranges. Rings B (C4-C6/C11-C13), C (C6-C11) and D (C15-C20) are, of course, planar and the dihedral angles between them are B/C = 1.33 (3)°, B/D = 83.55 (3)° and C/D = 82.65 (3)°. So, rings B and C are nearly coplanar. Ring A (O1/C1-C4/C13) is not planar, having total puckering amplitude, Q_T_, of 0.172 (3) and flattened-boat conformation [φ = -22.99 (3)° and θ = 105.077 (4)°] (Cremer & Pople, 1975).

In the crystal structure, intermolecular N-H···N and N-H···O hydrogen bonds (Table 1) generate edge-fused R_2_^2^(12) and R_2_^2^(14) ring motifs (Fig. 2) (Bernstein *et al.*,  1995). The hydrogen bonded motifs are linked to each other to form a three dimensional network, in which they may be effective in the stabilization of the structure. The π-π contact between the chlorophenyl rings, Cg4—Cg4^i^ [symmetry code: (i) -x, -y, 2 - z, where Cg4 is centroid of the ring D (C15-C20)] may further stabilize the structure, with centroid-centroid distance of 3.879 (3) Å.

### Experimental

The title compound was prepared by the reaction of 2-(2-chlorobenzylidene)- malononitrile (1 mmol) and 2-hydroxynaphthalene-1,4-dione (1 mmol) in glacial acetic acid without catalyst. Crystals suitable for X-ray analysis were obtained by slow evaporation of an aqueous ethanol solution (95%) (yield; 90%; m.p. > 573 K).

### Refinement

H atoms were positioned geometrically, with N-H = 0.86 Å (for NH_2_) and C-H = 0.93 and 0.98 Å for aromatic and methine H, respectively, and constrained to ride on their parent atoms with U_iso_(H) = 1.2U_eq_(C,N).

### Crystal data

**Table:**

C_20_H_11_ClN_2_O_3_	*Z* = 2
*M**_r_* = 362.76	*F*(000) = 372
Triclinic, *P*1	*D*_x_ = 1.517 Mg m^−^^3^
Hall symbol: -P 1	Melting point > 573 K
*a* = 8.3201 (10) Å	Mo *K*α radiation, λ = 0.71073 Å
*b* = 9.3729 (12) Å	Cell parameters from 959 reflections
*c* = 11.0081 (16) Å	θ = 2.8–25.1°
α = 93.015 (1)°	µ = 0.27 mm^−^^1^
β = 96.393 (1)°	*T* = 298 K
γ = 110.732 (2)°	Block, orange
*V* = 793.95 (18) Å^3^	0.17 × 0.15 × 0.10 mm

### Data collection

**Table:**

Bruker SMART CCD area-detector diffractometer	2746 independent reflections
Radiation source: fine-focus sealed tube	1566 reflections with *I* > 2σ(*I*)
graphite	*R*_int_ = 0.025
φ and ω scans	θ_max_ = 25.0°, θ_min_ = 1.9°
Absorption correction: multi-scan (*SADABS*; Sheldrick, 1996)	*h* = −9→9
*T*_min_ = 0.956, *T*_max_ = 0.974	*k* = −11→10
4207 measured reflections	*l* = −13→8

### Refinement

**Table:**

Refinement on *F*^2^	Primary atom site location: structure-invariant direct methods
Least-squares matrix: full	Secondary atom site location: difference Fourier map
*R*[*F*^2^ > 2σ(*F*^2^)] = 0.053	Hydrogen site location: inferred from neighbouring sites
*wR*(*F*^2^) = 0.100	H-atom parameters constrained
*S* = 1.04	*w* = 1/[σ^2^(*F*_o_^2^) + (0.0295*P*)^2^] where *P* = (*F*_o_^2^ + 2*F*_c_^2^)/3
2746 reflections	(Δ/σ)_max_ < 0.001
235 parameters	Δρ_max_ = 0.40 e Å^−^^3^
0 restraints	Δρ_min_ = −0.26 e Å^−^^3^

### Special details

**Table:**

Geometry. All e.s.d.'s (except the e.s.d. in the dihedral angle between two l.s. planes) are estimated using the full covariance matrix. The cell e.s.d.'s are taken into account individually in the estimation of e.s.d.'s in distances, angles and torsion angles; correlations between e.s.d.'s in cell parameters are only used when they are defined by crystal symmetry. An approximate (isotropic) treatment of cell e.s.d.'s is used for estimating e.s.d.'s involving l.s. planes.
Refinement. Refinement of *F*^2^ against ALL reflections. The weighted *R*-factor *wR* and goodness of fit *S* are based on *F*^2^, conventional *R*-factors *R* are based on *F*, with *F* set to zero for negative *F*^2^. The threshold expression of *F*^2^ > σ(*F*^2^) is used only for calculating *R*-factors(gt) *etc*. and is not relevant to the choice of reflections for refinement. *R*-factors based on *F*^2^ are statistically about twice as large as those based on *F*, and *R*- factors based on ALL data will be even larger.

### Fractional atomic coordinates and isotropic or equivalent isotropic displacement parameters (Å^2^)

**Table:**

	*x*	*y*	*z*	*U*_iso_*/*U*_eq_	
Cl1	−0.01619 (12)	0.04001 (10)	0.69113 (9)	0.0587 (3)	
O1	0.1440 (3)	0.3890 (2)	0.6133 (2)	0.0405 (6)	
O2	0.5886 (3)	0.2063 (3)	0.7464 (2)	0.0541 (7)	
O3	0.2251 (3)	0.3753 (3)	0.3898 (2)	0.0536 (7)	
N1	−0.0034 (3)	0.4822 (3)	0.7313 (2)	0.0471 (8)	
H1A	−0.0304	0.5099	0.7994	0.057*	
H1B	−0.0556	0.4936	0.6626	0.057*	
N2	0.1921 (4)	0.4871 (4)	1.0491 (3)	0.0578 (9)	
C1	0.1196 (4)	0.4216 (3)	0.7320 (3)	0.0369 (8)	
C2	0.2135 (4)	0.3957 (3)	0.8299 (3)	0.0344 (8)	
C3	0.3297 (4)	0.3028 (3)	0.8197 (3)	0.0336 (8)	
H3	0.4412	0.3622	0.8696	0.040*	
C4	0.3646 (4)	0.2940 (3)	0.6883 (3)	0.0309 (8)	
C5	0.5061 (4)	0.2424 (3)	0.6626 (3)	0.0360 (8)	
C6	0.5448 (4)	0.2361 (3)	0.5342 (3)	0.0339 (8)	
C7	0.6774 (4)	0.1889 (4)	0.5076 (3)	0.0466 (9)	
H7	0.7416	0.1604	0.5699	0.056*	
C8	0.7152 (5)	0.1837 (4)	0.3890 (3)	0.0538 (10)	
H8	0.8064	0.1540	0.3718	0.065*	
C9	0.6180 (4)	0.2225 (4)	0.2961 (3)	0.0530 (10)	
H9	0.6426	0.2170	0.2160	0.064*	
C10	0.4851 (4)	0.2690 (4)	0.3207 (3)	0.0461 (9)	
H10	0.4202	0.2951	0.2574	0.055*	
C11	0.4476 (4)	0.2770 (3)	0.4401 (3)	0.0346 (8)	
C12	0.3083 (4)	0.3326 (3)	0.4679 (3)	0.0350 (8)	
C13	0.2762 (4)	0.3374 (3)	0.5983 (3)	0.0337 (8)	
C14	0.1989 (4)	0.4470 (4)	0.9505 (3)	0.0405 (9)	
C15	0.2617 (4)	0.1487 (3)	0.8721 (3)	0.0322 (8)	
C16	0.1126 (4)	0.0269 (4)	0.8235 (3)	0.0374 (9)	
C17	0.0573 (4)	−0.1101 (4)	0.8767 (3)	0.0450 (9)	
H17	−0.0427	−0.1903	0.8412	0.054*	
C18	0.1515 (5)	−0.1260 (4)	0.9819 (3)	0.0538 (10)	
H18	0.1157	−0.2177	1.0179	0.065*	
C19	0.2985 (5)	−0.0068 (4)	1.0343 (3)	0.0514 (10)	
H19	0.3613	−0.0172	1.1065	0.062*	
C20	0.3530 (4)	0.1287 (4)	0.9798 (3)	0.0433 (9)	
H20	0.4532	0.2084	1.0158	0.052*	

### Atomic displacement parameters (Å^2^)

**Table:**

	*U*^11^	*U*^22^	*U*^33^	*U*^12^	*U*^13^	*U*^23^
Cl1	0.0512 (6)	0.0542 (6)	0.0649 (7)	0.0171 (4)	−0.0099 (5)	0.0082 (5)
O1	0.0409 (14)	0.0529 (14)	0.0391 (14)	0.0302 (12)	0.0069 (11)	0.0067 (12)
O2	0.0534 (16)	0.0759 (17)	0.0500 (16)	0.0423 (14)	0.0080 (13)	0.0191 (14)
O3	0.0643 (17)	0.0733 (17)	0.0398 (15)	0.0459 (14)	0.0020 (13)	0.0114 (13)
N1	0.0525 (19)	0.0636 (19)	0.0407 (18)	0.0403 (16)	0.0056 (15)	0.0031 (16)
N2	0.065 (2)	0.066 (2)	0.046 (2)	0.0297 (18)	0.0090 (18)	−0.0059 (18)
C1	0.037 (2)	0.0340 (19)	0.044 (2)	0.0177 (16)	0.0102 (18)	0.0047 (17)
C2	0.037 (2)	0.0356 (19)	0.035 (2)	0.0183 (16)	0.0072 (17)	0.0027 (17)
C3	0.0340 (19)	0.0366 (19)	0.033 (2)	0.0165 (15)	0.0015 (16)	0.0036 (17)
C4	0.0328 (19)	0.0285 (17)	0.034 (2)	0.0135 (15)	0.0057 (16)	0.0044 (16)
C5	0.0319 (19)	0.0350 (19)	0.044 (2)	0.0162 (16)	0.0015 (18)	0.0081 (18)
C6	0.033 (2)	0.0334 (18)	0.036 (2)	0.0121 (15)	0.0061 (17)	0.0041 (17)
C7	0.043 (2)	0.053 (2)	0.054 (3)	0.0278 (18)	0.0078 (19)	0.008 (2)
C8	0.049 (2)	0.065 (3)	0.055 (3)	0.028 (2)	0.016 (2)	−0.002 (2)
C9	0.051 (2)	0.065 (3)	0.044 (2)	0.021 (2)	0.010 (2)	−0.001 (2)
C10	0.043 (2)	0.054 (2)	0.044 (2)	0.0207 (18)	0.0074 (19)	0.003 (2)
C11	0.035 (2)	0.0324 (19)	0.035 (2)	0.0108 (15)	0.0047 (17)	−0.0009 (17)
C12	0.035 (2)	0.0325 (19)	0.039 (2)	0.0132 (15)	0.0047 (17)	0.0037 (17)
C13	0.0346 (19)	0.0297 (18)	0.040 (2)	0.0158 (15)	0.0061 (17)	0.0012 (17)
C14	0.039 (2)	0.037 (2)	0.048 (2)	0.0164 (16)	0.0055 (19)	0.008 (2)
C15	0.0331 (19)	0.0377 (19)	0.0338 (19)	0.0207 (16)	0.0098 (16)	0.0064 (17)
C16	0.036 (2)	0.042 (2)	0.042 (2)	0.0234 (16)	0.0058 (17)	0.0035 (18)
C17	0.041 (2)	0.036 (2)	0.062 (3)	0.0163 (16)	0.0148 (19)	0.0048 (19)
C18	0.062 (3)	0.050 (2)	0.063 (3)	0.031 (2)	0.021 (2)	0.023 (2)
C19	0.059 (3)	0.059 (3)	0.046 (2)	0.032 (2)	0.009 (2)	0.015 (2)
C20	0.045 (2)	0.047 (2)	0.040 (2)	0.0190 (17)	0.0035 (18)	0.0086 (18)

### Geometric parameters (Å, °)

**Table:**

Cl1—C16	1.747 (3)	C7—C8	1.379 (5)
O1—C1	1.380 (4)	C7—H7	0.9300
O1—C13	1.370 (3)	C8—C9	1.377 (4)
O2—C5	1.223 (3)	C8—H8	0.9300
O3—C12	1.216 (3)	C9—C10	1.372 (4)
N1—C1	1.334 (3)	C9—H9	0.9300
N1—H1A	0.8600	C10—C11	1.389 (4)
N1—H1B	0.8600	C10—H10	0.9300
N2—C14	1.144 (4)	C11—C12	1.482 (4)
C1—C2	1.343 (4)	C12—C13	1.490 (4)
C2—C14	1.420 (5)	C15—C16	1.383 (4)
C2—C3	1.522 (4)	C15—C20	1.393 (4)
C3—C4	1.510 (4)	C16—C17	1.387 (4)
C3—C15	1.524 (4)	C17—C18	1.370 (4)
C3—H3	0.9800	C17—H17	0.9300
C4—C13	1.335 (4)	C18—C19	1.373 (4)
C4—C5	1.470 (4)	C18—H18	0.9300
C5—C6	1.487 (4)	C19—C20	1.383 (4)
C6—C7	1.380 (4)	C19—H19	0.9300
C6—C11	1.396 (4)	C20—H20	0.9300
			
C13—O1—C1	117.4 (2)	C8—C9—H9	119.7
C1—N1—H1A	120.0	C9—C10—C11	120.0 (3)
C1—N1—H1B	120.0	C9—C10—H10	120.0
H1A—N1—H1B	120.0	C11—C10—H10	120.0
N1—C1—C2	127.8 (3)	C10—C11—C6	119.6 (3)
N1—C1—O1	110.1 (3)	C10—C11—C12	120.2 (3)
C2—C1—O1	122.1 (3)	C6—C11—C12	120.2 (3)
C1—C2—C14	120.4 (3)	O3—C12—C11	122.6 (3)
C1—C2—C3	122.9 (3)	O3—C12—C13	120.7 (3)
C14—C2—C3	116.6 (3)	C11—C12—C13	116.8 (3)
C4—C3—C2	108.4 (2)	C4—C13—O1	124.6 (3)
C4—C3—C15	114.8 (2)	C4—C13—C12	123.5 (3)
C2—C3—C15	113.2 (3)	O1—C13—C12	111.9 (3)
C4—C3—H3	106.6	N2—C14—C2	177.6 (4)
C2—C3—H3	106.6	C16—C15—C20	116.6 (3)
C15—C3—H3	106.6	C16—C15—C3	125.4 (3)
C13—C4—C5	120.3 (3)	C20—C15—C3	118.0 (3)
C13—C4—C3	121.8 (3)	C15—C16—C17	122.3 (3)
C5—C4—C3	117.8 (3)	C15—C16—Cl1	120.9 (2)
O2—C5—C4	119.6 (3)	C17—C16—Cl1	116.8 (3)
O2—C5—C6	121.8 (3)	C18—C17—C16	119.4 (3)
C4—C5—C6	118.6 (3)	C18—C17—H17	120.3
C7—C6—C11	119.7 (3)	C16—C17—H17	120.3
C7—C6—C5	119.8 (3)	C17—C18—C19	120.1 (3)
C11—C6—C5	120.6 (3)	C17—C18—H18	120.0
C8—C7—C6	120.2 (3)	C19—C18—H18	120.0
C8—C7—H7	119.9	C18—C19—C20	119.9 (3)
C6—C7—H7	119.9	C18—C19—H19	120.0
C9—C8—C7	120.0 (4)	C20—C19—H19	120.0
C9—C8—H8	120.0	C19—C20—C15	121.6 (3)
C7—C8—H8	120.0	C19—C20—H20	119.2
C10—C9—C8	120.5 (4)	C15—C20—H20	119.2
C10—C9—H9	119.7		
			
C13—O1—C1—N1	174.8 (2)	C7—C6—C11—C12	−178.1 (3)
C13—O1—C1—C2	−3.9 (4)	C5—C6—C11—C12	2.1 (4)
N1—C1—C2—C14	−5.9 (6)	C10—C11—C12—O3	−1.4 (5)
O1—C1—C2—C14	172.6 (3)	C6—C11—C12—O3	177.0 (3)
N1—C1—C2—C3	169.9 (3)	C10—C11—C12—C13	179.9 (3)
O1—C1—C2—C3	−11.5 (5)	C6—C11—C12—C13	−1.7 (4)
C1—C2—C3—C4	18.2 (4)	C5—C4—C13—O1	−179.1 (3)
C14—C2—C3—C4	−165.8 (3)	C3—C4—C13—O1	−2.0 (5)
C1—C2—C3—C15	−110.3 (4)	C5—C4—C13—C12	2.0 (5)
C14—C2—C3—C15	65.6 (4)	C3—C4—C13—C12	179.1 (3)
C2—C3—C4—C13	−11.5 (4)	C1—O1—C13—C4	11.0 (4)
C15—C3—C4—C13	116.1 (3)	C1—O1—C13—C12	−170.1 (3)
C2—C3—C4—C5	165.6 (3)	O3—C12—C13—C4	−179.1 (3)
C15—C3—C4—C5	−66.7 (3)	C11—C12—C13—C4	−0.4 (4)
C13—C4—C5—O2	178.4 (3)	O3—C12—C13—O1	1.9 (4)
C3—C4—C5—O2	1.1 (4)	C11—C12—C13—O1	−179.4 (2)
C13—C4—C5—C6	−1.6 (4)	C4—C3—C15—C16	−57.8 (4)
C3—C4—C5—C6	−178.8 (3)	C2—C3—C15—C16	67.4 (4)
O2—C5—C6—C7	−0.2 (5)	C4—C3—C15—C20	124.2 (3)
C4—C5—C6—C7	179.7 (3)	C2—C3—C15—C20	−110.6 (3)
O2—C5—C6—C11	179.5 (3)	C20—C15—C16—C17	−1.4 (5)
C4—C5—C6—C11	−0.6 (4)	C3—C15—C16—C17	−179.4 (3)
C11—C6—C7—C8	0.7 (5)	C20—C15—C16—Cl1	178.3 (2)
C5—C6—C7—C8	−179.5 (3)	C3—C15—C16—Cl1	0.2 (4)
C6—C7—C8—C9	−1.4 (5)	C15—C16—C17—C18	0.8 (5)
C7—C8—C9—C10	1.1 (5)	Cl1—C16—C17—C18	−178.8 (3)
C8—C9—C10—C11	−0.1 (5)	C16—C17—C18—C19	0.4 (5)
C9—C10—C11—C6	−0.6 (5)	C17—C18—C19—C20	−1.0 (5)
C9—C10—C11—C12	177.8 (3)	C18—C19—C20—C15	0.4 (5)
C7—C6—C11—C10	0.3 (5)	C16—C15—C20—C19	0.8 (5)
C5—C6—C11—C10	−179.5 (3)	C3—C15—C20—C19	179.0 (3)

### Hydrogen-bond geometry (Å, °)

**Table:**

*D*—H···*A*	*D*—H	H···*A*	*D*···*A*	*D*—H···*A*
N1—H1A···N2^i^	0.86	2.26	3.080 (4)	159
N1—H1B···O3^ii^	0.86	2.22	2.889 (3)	134

Symmetry codes: (i) −*x*, −*y*+1, −*z*+2; (ii) −*x*, −*y*+1, −*z*+1.

## References

[bb1] Allen, F. H., Kennard, O., Watson, D. G., Brammer, L., Orpen, A. G. & Taylor, R. (1987). *J. Chem. Soc. Perkin Trans. 2*, pp. S1–19.

[bb2] Banzatti, C., Branzoli, U., Lovisolo, M. P., Melloni, P. & Salvador, P. (1984). *Arzneim. Forsch.* **34**, 864–869. 6541922

[bb3] Bernstein, J., Davis, R. E., Shimoni, L. & Chang, N.-L. (1995). *Angew. Chem. Int. Ed. Engl.* **34**, 155–1573.

[bb4] Bruker (1997). *SMART* and *SAINT* Bruker AXS Inc., Madison, Wisconsin, USA.

[bb5] Cremer, D. & Pople, J. A. (1975). *J. Am. Chem. Soc.* **97**, 1354–1358.

[bb6] El-Agrody, A. M., El-Hakim, M. H., Abd El-Latif, M. S., Fakery, A. H., El-Sayed, E. S. M. & El-Ghareab, K. A. (2000). *Acta Pharm.* **50**, 111–120.

[bb7] Hatakeyama, S., Ochi, N., Numata, H. & Takano, S. (1988). *J. Chem. Soc. Chem. Commun.* pp. 1202–1204.

[bb8] Kongkathip, N., Kongkathip, B., Siripong, P., Sangma, C., Luangkamin, S., Niyomdecha, M., Pattanapa, S., Piyaviriyagul, S. & Kongsaeree, P. (2003). *Bioorg. Med. Chem.* **11**, 3179–3191. 10.1016/s0968-0896(03)00226-8 12818681

[bb9] Mohr, S. J., Chirigos, M. A., Fuhrman, F. S. & Pryor, J. W. (1975). *Cancer Res.* **35**, 3750–3754. 1192431

[bb10] Sheldrick, G. M. (1996). *SADABS* University of Göttingen, Germany.

[bb11] Sheldrick, G. M. (2008). *Acta Cryst.* A**64**, 112–122. 10.1107/S0108767307043930 18156677

[bb12] Spek, A. L. (2003). *J. Appl. Cryst.* **36**, 7–13.

[bb13] Tandon, V. K., Vaish, M., Jain, S., Bhakuni, D. S. & Srimal, R. C. (1991). *Indian J. Pharm. Sci.* **53**, 22–23.

